# The Role of Mitochondrial Damage-Associated Molecular Patterns in Chronic Neuroinflammation

**DOI:** 10.1155/2019/4050796

**Published:** 2019-04-01

**Authors:** Ekta Bajwa, Caitlin B. Pointer, Andis Klegeris

**Affiliations:** Department of Biology, University of British Columbia Okanagan Campus, Kelowna, BC, Canada

## Abstract

Mitochondrial dysfunction has been established as a common feature of neurodegenerative disorders that contributes to disease pathology by causing impaired cellular energy production. Mitochondrial molecules released into the extracellular space following neuronal damage or death may also play a role in these diseases by acting as signaling molecules called damage-associated molecular patterns (DAMPs). Mitochondrial DAMPs have been shown to initiate proinflammatory immune responses from nonneuronal glial cells, including microglia and astrocytes; thereby, they have the potential to contribute to the chronic neuroinflammation present in these disorders accelerating the degeneration of neurons. In this review, we highlight the mitochondrial DAMPs cytochrome *c* (CytC), mitochondrial transcription factor A (TFAM), and cardiolipin and explore their potential role in the central nervous system disorders including Alzheimer's disease and Parkinson's disease, which are characterized by neurodegeneration and chronic neuroinflammation.

## 1. Introduction

Neurodegenerative diseases are a heterogenous group of chronic disorders characterized by gradual and progressive loss or dysfunction of select neuronal subtypes. Prototypical examples include Alzheimer's disease (AD), Parkinson's disease (PD), amyotrophic lateral sclerosis, and Huntington's disease. Over the past several decades, evidence has accumulated suggesting that impaired mitochondrial function is a common mechanism in these diseases [1]. Mitochondria are cytoplasmic organelles that undertake vital metabolic and cellular functions, the most important of which is the generation of adenosine triphosphate (ATP) by oxidative phosphorylation. In addition, mitochondria play a central role in apoptosis and intracellular calcium homeostasis as well as the production and modulation of reactive oxygen species (ROS) [2]. Mitochondria have a semiautonomous genome represented by mitochondrial DNA (mtDNA) that codes for some of the structural components of the respiratory chain required for ATP production and the machinery required for intramitochondrial protein synthesis [3]. Mitochondrial abnormalities that have been identified in neurodegenerative diseases include mtDNA mutations that result in respiratory chain deficiencies, increased generation of ROS causing oxidative stress, and changes in mitochondrial structure, all of which lead to improper mitochondrial function and impaired energy production [4–8]. Considering the high metabolic demand of the central nervous system (CNS) and the limited regenerative capacity of neurons, mitochondrial dysfunction can be detrimental to all brain cell types and contribute to neuronal death.

Chronic inflammation is another characteristic of neurodegenerative diseases mediated by nonneuronal glial cells, including microglia and astrocytes [9]. Microglia are a distinct population of mononuclear phagocytes that represent the innate immune cells of the brain [10]. Upon recognition of foreign pathogens or other damaging stimuli, microglia attempt to restore the homeostatic conditions of the CNS by undergoing a process of activation characterized by alterations in their secretory profile, morphology, and phagocytic activity [11, 12]. Microglial activation is associated with increased expression of the inducible nitric oxide synthase (iNOS) and nicotinamide adenine dinucleotide phosphate (NADPH) oxidase enzymes, which are responsible for generating cytotoxic nitrogen and oxygen species [13, 14]. Additionally, microglial activation is accompanied by the secretion of proinflammatory cytokines such as interleukin (IL)-1*β*, IL-6, and tumor necrosis factor (TNF)-*α* [15–18]. The nonspecific nature of the inflammatory response of activated microglia may also induce collateral damage of the surrounding cells including neurons and other glial cells. The damaged or dying cells that result from such deleterious conditions release endogenous molecules termed damage-associated molecule patterns (DAMPs) capable of causing adverse glial cell activation, creating a self-propagating cycle of neuroinflammation, and accelerating cell death [19–21]. Under the pathological conditions present in neurodegenerative disorders, microglia are chronically and adversely activated thereby mediating the neuroinflammatory processes that could contribute to the degeneration of neurons [10].

DAMPs were first described as molecules released during necrotic cell death following traumatic injury or prolonged inflammation. They alert the immune system to danger [20]. Under physiological conditions, DAMPs are sequestered intracellularly where they perform distinct functions contributing to cellular homeostasis; however, upon loss of membrane integrity and their release into the extracellular environment, they confer proinflammatory roles aimed at inducing and facilitating the repair of host tissue [22, 23]. The immune response triggered by DAMPs is termed “sterile inflammation,” as it occurs in the absence of infectious agents [24]. Through their interaction with pattern recognition receptors (PRRs) and other immune cell receptors, DAMPs prime the process of antigen presentation by dendritic cells and induce the expression of proinflammatory molecules and nitric oxide (NO) in peripheral macrophages and microglia of the CNS [19, 25, 26].

**Figure 1:**
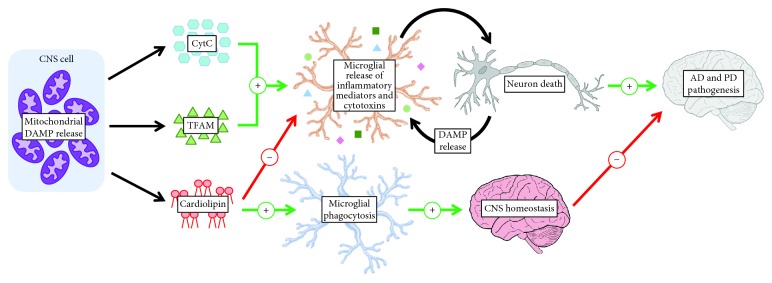
Mitochondrial damage-associated molecular patterns (DAMPs) cytochrome *c* (CytC), mitochondrial transcription factor A (TFAM), and cardiolipin can be released into the central nervous system (CNS) intercellular space where they regulate microglial phagocytosis and the release of inflammatory mediators by microglia. AD = Alzheimer's disease; PD = Parkinson's disease.

Recent studies have revealed the mitochondria as organelles that store and release several specific DAMPs [27, 28]. According to the “endosymbiont hypothesis,” mitochondria evolved from *α*-proteobacteria that entered a symbiotic relationship with an ancestral eukaryotic cell [29]. Due to their endosymbiotic origin, mitochondria possess bacterial features such as circular double-stranded mtDNA containing cytosine phosphate guanosine (CpG), N-formylated peptides, and the membrane lipid cardiolipin [27]. As a result of the similarities between mitochondrial and bacterial molecules, several distinct mitochondrial components are capable of engaging PRRs that recognize bacterial structures [30]. Therefore, an emerging role of mitochondria in the pathogenesis of neurodegenerative disorders involves harboring and releasing molecules that can contribute to neurotoxic inflammatory environments. In this review, we will be highlighting three mitochondrial components capable of serving as DAMPs once they have been released extracellularly within the CNS: the mitochondrial respiratory chain protein cytochrome *c* (CytC), mitochondrial transcription factor A (TFAM), and the mitochondrial membrane lipid cardiolipin (Figure 1).

## 2. Cytochrome *c* (CytC)

CytC is a 12 kDA mitochondrial protein comprised of 104 amino acids and a single covalently attached heme group [31]. CytC is primarily found in the mitochondrial intermembrane space, where it functions as an electron carrier in the electron transport chain (ETC) and as a scavenger of ROS [32, 33]. During cellular stress, such as heat or oxidative damage, CytC also exhibits peroxidase activity leading to the oxidation of the mitochondrial membrane lipid cardiolipin, which is required for membrane permeabilization and release of CytC into the cytosol [34]. Within the cytosol, CytC acts as a key initiator of apoptosis by facilitating the assembly of apoptosomes that activate the caspase cascade leading to cellular death [35]. Considering its critical role in mediating the apoptotic pathway, CytC has been implicated in the pathogenesis of diseases characterized by excessive cell death including the neurodegenerative diseases AD and PD [35, 36]. The mitochondrial dysfunction present in neurodegenerative pathologies increases the permeability of the mitochondrial membrane, which may lead to increased cytosolic release of CytC and subsequent apoptosis of neurons and nonneuronal glial cells [37–39].

CytC may also contribute to the progression of neurodegenerative diseases by acting extracellularly as a DAMP and amplifying the neuroinflammatory state that is one of the characteristics of these pathologies. CytC has emerged as a potential DAMP due to the discovery of its elevated extracellular concentration following cell death in the periphery as well as in the CNS. Damaged neurons have been shown to release CytC into cell culture medium [40]. The release of CytC into the extracellular space by splenocytes was observed immediately following the induction of cellular necrosis, and more delayed release followed apoptotic insults [41]. Following myocardial infarction or traumatic brain injury, CytC concentrations reached 4.6 *μ*g/ml in the serum and were as high as 50 ng/ml in the cerebrospinal fluid, representing a 50-fold increase over baseline levels [42, 43]. High extracellular concentrations of CytC in both these pathologies correlated with poor clinical outcomes.

Several immunomodulatory properties of extracellular CytC have been observed in the periphery, which are similar to those of other prototypical DAMPs. For example, intra-articular injection of CytC in mice resulted in the accumulation of neutrophils and macrophages within synovial joints causing damage similar to the chronic inflammation in rheumatoid arthritis (RA) leading to the development of RA-like symptoms. In addition, CytC stimulation of mouse splenocytes caused an activation of nuclear factor (NF)-*κ*B accompanied by the release of proinflammatory mediators IL-6, TNF-*α*, macrophage inflammatory protein (MIP)-1*α*, MIP-2, and monocyte chemoattractant protein (MCP)-1 [44]. Primary lymphocytes and CD8+ dendritic cells underwent apoptosis following exposure to extracellular CytC [45, 46]. These studies demonstrated that the soluble serum protein alpha-2-leucine-rich-glycoprotein inhibited CytC-induced cytotoxicity by binding to and scavenging CytC from the extracellular space, which implies that there are endogenous proteins that function to protect against the cytotoxicity and inflammation caused by extracellular CytC, which is released following significant tissue damage [45].

Studies have been performed demonstrating the potential for extracellular CytC to act as a DAMP within the CNS and modulate glial cell immune responses. Gouveia et al. [47] conducted *in vitro* experiments demonstrating that the addition of CytC in combination with lipopolysaccharide (LPS) to phorbol 12-myristate 13-acetate (PMA)-differentiated THP-1 monocytic cells, which were used as a model for human microglia, led to a neurotoxic inflammatory response. This study also demonstrated enhanced release of specific cytotoxins by microglia-like cells in response to CytC stimulation. For example, CytC was found to prime the NADPH oxidase-dependent respiratory burst of dimethyl sulfoxide (DMSO)-differentiated promyelocytic HL-60 cells leading to increased ROS production; this cellular effect of CytC was enhanced in the presence of interferon (IFN)-*γ*. Additionally, CytC exacerbated the secretion of NO by IFN-*γ*-activated murine BV-2 microglial cells [47]. The synergistic effects of CytC and other inflammatory stimuli are also observed for other DAMPs, such as TFAM, and suggest that the interplay between DAMPs and other inflammatory signals may occur under neuroinflammatory conditions [48, 49]. The release of ROS and reactive nitrogen species (RNS) could be beneficial to the maintenance of homeostatic conditions by eliminating foreign substances and pathogens; however, increased microglial production of these cytotoxins in response to extracellularly released CytC may contribute to the oxidative and nitrosative stress observed in neurodegenerative disorders that lead to neuronal death [50, 51]. Thus, CytC that is released extracellularly in the CNS may be recognized by microglia and lead to neuroinflammatory responses that are detrimental to the survival of neurons (see Figure 1); however, the extracellular effects of CytC on glial cells are yet to be confirmed *in vivo*.

Immune cell recognition of DAMPs occurs through PRRs, which also engage conserved pathogen-associated molecular patterns (PAMPs) present on invading microbes and viruses [52]. Toll-like receptors (TLRs) represent a family of PRRs that have been shown to initiate immune responses upon interacting with several different DAMPs [20]. However, DAMP-mediated immune responses in some cases still occur in the absence of TLR signaling. Other receptors engaged by DAMPs include nucleotide-binding oligomerization domain-like receptors, formyl peptide receptors, retinoic acid-inducible gene 1-like receptors, and the receptor for advanced glycation end products (RAGE) [53–56]. To elucidate the mechanisms mediating the effects of extracellular CytC as a DAMP, Gouveia et al. [47] assessed microglial responses to extracellular CytC exposure *in vitro* following inhibition of candidate receptors and intracellular signaling pathways. They showed that the production of ROS by CytC-primed HL-60 cells was significantly reduced by blocking TLR4, but not RAGE, suggesting that the interaction between CytC and microglia leading to proinflammatory effects may occur at least partially through TLR4 [47].

The central event in many DAMP-mediated immune responses is the activation of mitogen-activated protein kinase (MAPK) signaling cascades, which act downstream of PRRs [54]. The Jun N-terminal kinase (JNK) pathway is known to be activated following TLR4 binding and has also been implicated in the production of RNS [57]. Gouviea et al. [47] demonstrated that pretreatment of murine BV-2 microglial cells with a specific inhibitor of the JNK pathway (SP600125) attenuated the NO secretion induced by CytC. These results indicate that the function of CytC as a DAMP may be dependent on the activation of the JNK pathway similar to the mitochondrial DAMP TFAM and the well-characterized DAMP S100B [48, 58].  Table 1 summarizes the extracellular effects of CytC.

## 3. Mitochondrial Transcription Factor A (TFAM)

TFAM is a 25 kDa protein abundant in the mitochondria where it regulates gene transcription and maintains mtDNA structure [59, 60]. TFAM binds to a 22-base-pair region of mtDNA upstream from promoter sites and causes a turn in the DNA exposing a specific binding site for transcription machinery upstream from the transcription initiation site [61, 62]. TFAM consists of 246 amino acids arranged into several well-defined domains including two high-mobility group box (HMGB) domains (Box A and Box B), which make it a member of the highly conserved and ubiquitous HMGB family of DNA-binding proteins [59]. HMGB1 is arguably the best characterized member of the HMGB family of proteins. It is a nuclear DNA-binding protein that exhibits approximately 76% sequence homology with TFAM [63]. HMGB1 is expressed by almost all vertebrate cells, and upon its release into the extracellular space, it is capable of initiating inflammatory responses by activating several different cell types [64]. Within the CNS, HMGB1 is released by damaged cells as well as actively secreted by neurons and glia. HMGB1 has been shown to function as a proinflammatory mediator that can cause microglial activation characterized by the release of cytokines such as TNF-*α* and IL-1*β* as well as the chemokine MCP-1 [65–67]. Considering that TFAM is a structural and functional homolog of HMGB1, its extracellular role as a potential DAMP has been implicated. Under physiological conditions, TFAM is localised to the inner mitochondrial membrane; however, upon cellular damage, TFAM can also be released into the extracellular environment where it can function as a proinflammatory signaling molecule in a manner similar to HMGB1 [68–70]. In the peripheral tissues, the combination of TFAM and N-formyl peptides has been shown to trigger the release of IL-8 from human peripheral blood monocytes [70]. Another study observed that TFAM augmented plasmacytoid dendritic cell activation induced by CpG DNA, which was characterized by increased release of TNF-*α*. TFAM in combination with CpG DNA was also shown to induce TNF-*α* secretion by human splenocytes [71].

The proinflammatory activity of extracellular TFAM demonstrated in peripheral tissues suggests that it could also play a role in the CNS inflammation observed in neurodegenerative diseases. Little et al. [48] reported that TFAM applied at low *μ*g/ml concentrations to human microglia-like THP-1 monocytic cells and peripheral blood monocytes increased their expression of IL-1*β*, IL-6, and IL-8. TFAM, in combination with IFN-*γ*, also induced the secretion of IL-6 by human microglia. Additionally, TFAM augmented the release of cytotoxins by IFN-*γ*-activated THP-1 cells as observed by the decreased viability of human SH-SY5Y neuronal cells exposed to supernatants from these monocytic cells [48]. IFN-*γ* is a proinflammatory molecule involved in regulating glial cell activation that is elevated in brains with neurodegenerative disease [72]. Sha et al. [73] suggested that binding of HMGB1 to inflammatory cytokines may be required for its proinflammatory activity; similarly, the proinflammatory activity of TFAM may require the formation of an immune complex with IFN-*γ*. Interestingly, TFAM in combination with IFN-*γ* increased the secretion of MCP-1 by THP-1 monocytic cells compared to TFAM alone, consistent with the above studies [74]. MCP-1 is a chemotactic factor that may contribute to the neurotoxic inflammatory response induced by TFAM through recruiting and activating the surrounding microglia, which express the MCP-1 receptor [75, 76]. Schindler et al. [74] also investigated the cellular mechanisms mediating the proinflammatory function of TFAM by selectively inhibiting receptors already implicated in HMGB1 signaling due to the structural similarity between these two DAMPs. Blocking the macrophage-1 antigen (Mac-1) and RAGE receptors using specific antibodies led to a reduction in MCP-1 secretion by THP-1 cells, suggesting that Mac-1 and RAGE may be partially engaged by TFAM and mediate its DAMP-like function similar to HMGB1 [74].

Microparticles (MPs) are intercellular signaling agents recently implicated in the progression of neurodegenerative diseases. They may also play a role in the neuroinflammatory processes induced by DAMPs [77]. MPs are submicron fragments released upon plasma membrane budding from various cell types including monocytes, microglia, astrocytes, and neurons [78–80]. The content of MPs can be transferred to target cells and include biological signals that differ based on the donor cell-type as well as the inducing stimulus [81]. MPs can regulate various biological processes including cell-cell interactions, cell proliferation, and inflammation [82]. TFAM was shown to stimulate the significant release of MPs by THP-1 monocytic cells compared to unstimulated cells. Moreover, the MPs released by THP-1 cells in response to TFAM were able to activate THP-1 cells in an autocrine manner and led to cytotoxicity as shown by a decrease in SH-SY5Y neuronal cell viability following exposure to supernatant from MP-stimulated monocytic cells. The MPs derived from TFAM-stimulated THP-1 cells also induced the secretion of MCP-1 by THP-1 cells [83]. These results indicate that TFAM may act as a DAMP that can activate microglial cells to release MPs that possess neurotoxic and inflammatory properties and could contribute to neurodegenerative disease pathology.

Studies have been performed to determine the ability of TFAM to activate microglia and induce neuroinflammation *in vivo* providing further insight into the role of TFAM as a DAMP within the CNS. Schindler et al. [74] demonstrated that TFAM injected into the cisterna magna of male Sprague-Dawley rats triggered neuroinflammation in the hippocampus and frontal cortex, which are the predominant brain areas affected by neurodegeneration in AD. Injected TFAM upregulated the expression of MCP-1, IL-1*β*, IL-6, and TNF-*α* in the hippocampus, the expression of MCP-1, IL-1*β*, and TNF-*α* in the frontal cortex, and the concentration of IL-1*β* in both brain regions. Extracellular TFAM applied in a concentration-dependent manner to isolated rat microglia upregulated the expression of inflammatory cytokines IL-6, IL-1*β*, and TNF-*α*, suggesting that microglia may be responsible for the neuroimmune response induced by TFAM *in vivo* [74].

Overall, the studies investigating the extracellular role of TFAM support its role as a DAMP that is capable of activating brain microglia leading to proinflammatory and neurotoxic responses (Table 2). TFAM, and the cellular receptors it engages, may represent novel targets for the development of therapeutic strategies against CNS pathologies characterized by neuroinflammation.

## 4. Cardiolipin

Cardiolipin is an anionic phospholipid found almost exclusively within the inner mitochondrial membrane of mammalian cells. Cardiolipin is essential for maintaining mitochondrial functioning and regulating several cellular metabolic processes [84, 85]. The specific functions of cardiolipin are supported by its unique structure, which includes a double glycerophosphate backbone and four fatty acid side chains. This is different from most other membrane phospholipids, which possess a single glycerophosphate backbone and two fatty acid side chains [86–88]. It has been proposed that, due to its distinctive structure, cardiolipin acquires a conical shape within the lipid bilayer, which contributes to its ability to promote electron transport efficiency, interact with proteins located both inside and outside of the mitochondria, and regulate mitochondrial membrane fusion and fission [87, 89–91].

The role of cardiolipin in regulating metabolic processes within healthy cells has been studied extensively; however, recent studies have implicated cardiolipin in pathologies involving damage or death of CNS cells [92, 93]. For example, cardiolipin has been implicated in the regulation of mitophagy, the elimination of dysfunctional mitochondria via autophagic processes, as well as apoptotic cell death [85, 94–96]. During these cellular processes, cardiolipin is redistributed from the inner mitochondrial membrane to the outer mitochondrial membrane by the mitochondrial isoform of the creatine kinase (MtCK) enzyme [97–99]. Redistribution of cardiolipin is a critical step in mitophagy, as it promotes the interaction of cardiolipin with several proteins involved in the initiation and propagation of this process, including microtubule-associated-protein-1-light chain-3 (LC3), which regulates mitochondrial engulfment and degradation via autophagosome formation [94, 99]. Furthermore, the redistribution of cardiolipin to the outer mitochondrial membrane allows cardiolipin to interact with various cell death-related proteins, including CytC and truncated BH3 interacting-domain death agonist (tBid), which are essential components of programmed apoptotic cell death [97, 100–102]. As a molecule expressed on the outer mitochondrial membrane, cardiolipin participates in the destruction of malfunctioning mitochondria, which is an essential process for maintaining homeostatic conditions in the CNS [96, 98, 103].

In addition to its roles in mitophagy and apoptosis, cardiolipin has also been implicated in regulating immune cell functions within the peripheral tissues and the CNS. Although primarily found within the mitochondria, cardiolipin can also be redistributed to the plasma membrane of a cell and be released extracellularly. More specifically, studies have shown that cardiolipin, as well as cardiolipin-containing mitochondria, can be relocated or released during cellular processes, such as apoptotic events, necrosis, and acute trauma, as well as in disease states [92, 104–107]. For example, it has been demonstrated that in mice afflicted with bacterial pneumonia, levels of extracellular cardiolipin are significantly increased within the lungs, which subsequently results in reduced immune cell production of IL-10, a cytokine involved in suppressing excessive inflammatory responses [107, 108]. Additionally, externalized cardiolipin has been shown to upregulate the phagocytic activity of the surrounding immune cells. Balasubramanian et al. [92] demonstrated that extracellular mitochondria with cardiolipin present on their surface can increase the phagocytic activity of peripheral macrophages by up to four times. Results from our laboratory showed that extracellular cardiolipin significantly upregulated the phagocytic activity of primary microglia isolated from C57BL/6 mouse brains. Extracellular cardiolipin also decreased the release of select proinflammatory mediators and cytotoxins including TNF-*α*, NO, and ROS from microglia-like cells [109]. These observations indicate that, following its release from cells, cardiolipin may act as a DAMP by regulating cytokine release and phagocytic activity of the surrounding immune cells, including microglia in the CNS [107, 109–112].

Although important in regulating homeostatic conditions, dysfunctional cardiolipin has been implicated in neurodegenerative disorders characterized by neurotoxic inflammatory states, such as AD and PD [93, 113, 114]. More specifically, it has been demonstrated that aging brains, particularly those affected by AD and PD, have significantly lower levels of cardiolipin, as well as increased ROS production. Such elevated oxidative stress results in excessive peroxidation of cardiolipin, which can lead to substantial modification of its structure [89, 115–117]. Although the exact mechanism by which peroxidation of cardiolipin occurs is not fully understood, its structural alteration can negatively affect phospholipid-protein interactions and promote membrane lipid degradation, which hinders cardiolipin's ability to effectively regulate mitochondrial functions [118–120]. The mitochondrial abnormalities associated with altered cardiolipin structure include decreased respiratory chain efficiency, impaired energy production, and excessive ROS production, which lead to a further increase in oxidative stress and contribute to the extensive cell death observed in AD and PD brains [85, 86, 89, 121]. Additionally, it has been demonstrated that decreased levels of cardiolipin are associated with CytC destabilization, reduced release of CytC from the mitochondria, and disrupted interactions between cardiolipin and MtCK or tBid, all of which hinder effective mitophagic and apoptotic processes [95, 103, 122]. Therefore, it is evident that preserving the levels and structure of intracellular cardiolipin is crucial for maintaining its functions as a regulator of CNS homeostasis. Meanwhile, extracellularly released cardiolipin may act as a DAMP by regulating neuroimmune responses (Table 3). In both capacities, cardiolipin appears to be an important regulator of the chronic neuroinflammatory state observed in neurodegenerative diseases, including AD and PD.

## 5. Conclusion

Due to the emerging role of impaired mitochondrial functioning in the pathogenesis of degenerative brain diseases, including AD, PD, amyotrophic lateral sclerosis, and Huntington's disease, it is critical to fully understand the roles mitochondrial molecules play in the inflammatory processes associated with these disorders. The purpose of this review was to highlight CytC, TFAM, and cardiolipin and their effects as DAMPs on CNS functioning and pathology. By further understanding the mechanisms through which these molecules act, therapeutic targets may be revealed for the treatment of neurodegenerative disorders characterized by mitochondrial dysfunction, neuroinflammation, and extensive cell death.

## Figures and Tables

**Table 1:** Effects of extracellular CytC on different cell types and signaling mechanisms activated in the target cells.

Target cell/tissue	Effects	Signaling receptor	Signaling pathway
Murine splenocytes	Release of IL-6, TNF-*α*, MIP-1*α*, MIP-2, and MCP-1 [44]		
Primary human lymphocytes	Induction of apoptosis [45]		
Murine CD8+ dendritic cells	Induction of apoptosis [46]		
Differentiated human promyelocytic HL-60 cells	Priming the NADPH oxidase-dependent respiratory burst leading to increased ROS production [47]	TLR4, but not RAGE, blockade decreases ROS release [47]	
Murine BV-2 microglial cells	Enhanced secretion of NO from cells activated by IFN-*γ* [47]		Inhibition of JNK, but not extracellular signal-regulated kinase (ERK) or p38 MAPK, pathways decreases NO secretion [47]
Human THP-1 monocytic cells	Costimulation with LPS induces cytotoxicity towards human SH-SY5Y neuronal cells [47]		
Murine knee joint	Enhanced neutrophil and macrophage accumulation in synovial fluid leading to histopathological and clinical signs of rheumatoid arthritis [44]		

**Table 2:** Effects of extracellular TFAM on different cell types and signaling mechanisms activated in the target cells.

Target cell/tissue	Effects	Signaling receptor	Signaling pathway
Human splenocytes	Costimulation with CpG DNA induces TNF-*α* secretion [71]		
Murine plasmacytoid dendritic cells	Costimulation with CpG DNA induces TNF-*α* secretion [69]	Blockade of TLR9 and RAGE decreases TNF-*α* secretion [71]	Activation of NF-*κ*B [69]
Human peripheral blood monocytes	Increased expression of IL-1*β*, IL-6, and IL-8 [48] Costimulation with N-formyl peptides induces IL-8 release [70]		
Human THP-1 monocytic cells	Costimulation with IFN-*γ* increases secretion of MCP-1 [74] Costimulation with IFN-*γ* induces release of cytotoxins leading to reduced viability of SH-SY5Y neuronal cells [48] Increased expression of IL-1*β*, IL-6, and IL-8 [48] Enhanced release of microparticles capable of autocrine induction of MCP-1 secretion as well as cytotoxicity towards SH-SY5Y neuronal cells [83]	Blockade of Mac-1 receptor and RAGE decreases MCP-1 secretion [74]	Inhibition of the JNK, but not p38 MAPK, pathway reduces cytotoxicity towards SH-SY5Y neuronal cells [48]
Human microglia	Costimulation with IFN-*γ* induces IL-6 secretion [48]		
Rat microglia	Upregulated expression of IL-6, IL-1*β*, and TNF-*α* [74]		
Rat hippocampus following injection into the cisterna magna	Upregulated expression of MCP-1, IL-1*β*, IL-6, and TNF-*α* and increased IL-1*β* concentration [74]		
Rat frontal cortex following injection into the cisterna magna	Upregulated expression of MCP-1, IL-1*β*, and TNF-*α* and increased IL-1*β* concentration [74]		

**Table 3:** Effects of extracellular cardiolipin on different cell types.

Target cell	Effect
Murine RAW 264.7 macrophages	Upregulated phagocytic activity [92]
Primary murine microglia	Upregulated phagocytic activity [109]
Human THP-1 monocytic cells	Attenuated IFN-*γ*-induced secretion of TNF-*α* [109]
Murine BV-2 microglial cells	Attenuated LPS-induced release of NO [109]
Human promyelocytic HL-60 cells	Attenuated LPS-primed NADPH oxidase-dependent respiratory burst leading to decreased ROS release [109]
